# The Effect of Framing and Normative Messages in Building Support for Climate Policies

**DOI:** 10.1371/journal.pone.0114335

**Published:** 2014-12-15

**Authors:** Mark J. Hurlstone, Stephan Lewandowsky, Ben R. Newell, Brittany Sewell

**Affiliations:** 1 School of Psychology, University of Western Australia, Crawley, Australia; 2 Centre for Environment and Life Sciences, Commonwealth Scientific and Industrial Research Organisation, Floreat, Australia; 3 Department of Experimental Psychology, University of Bristol, Bristol, United Kingdom; 4 School of Psychology, University of New South Wales, Sydney, Australia; Universidade de Vigo, Spain

## Abstract

Deep cuts in greenhouse gas emissions are required to mitigate climate change. However, there is low willingness amongst the public to prioritise climate policies for reducing emissions. Here we show that the extent to which Australians are prepared to reduce their country's CO_2_ emissions is greater when the costs to future national income are framed as a “foregone-gain”—incomes rise in the future but not by as much as in the absence of emission cuts—rather than as a “loss”—incomes decrease relative to the baseline expected future levels (Studies 1 & 2). The provision of a normative message identifying Australia as one of the world's largest CO_2_ emitters did not increase the amount by which individuals were prepared to reduce emissions (Study 1), whereas a normative message revealing the emission policy preferences of other Australians did (Study 2). The results suggest that framing the costs of reducing emissions as a smaller increase in future income and communicating normative information about others' emission policy preferences are effective methods for leveraging public support for emission cuts.

## Introduction

The average temperature of the Earth has been steadily increasing since the Industrial Revolution. The scientific consensus is that greenhouse gas (GHG) emissions from human agricultural and industrial activity are the principal cause of this global warming [1]–[3] and that such emissions must be severely curtailed to prevent further anthropogenic disruption of the climate system [4]. A significant barrier to achieving this goal is that many people are opposed to policies aimed at reducing GHG emissions [5]–[8]. The reasons for this resistance are numerous but one pivotal factor may be loss aversion [9]–[11]—people's powerful tendency to minimise losses—which may cause them to weight the immediate financial losses associated with reducing emissions more heavily than the potential future gains. Loss aversion, in turn, may trigger a preference towards maintaining the status quo [12] since the costs of departing from it loom larger than the potential benefits. There are also indications that a significant minority of people have an inflated estimation of the costs of reducing emissions—25% of Australians incorrectly think that reducing emissions will cause future incomes to decrease below current levels, rather than increase more slowly [13]—a belief which may exacerbate these cognitive biases.

One solution to counteracting these biases may lie in how messages about the costs of reducing emissions are formulated. It is well known that people's judgements and decisions are influenced by the way a problem is framed; that is, different but objectively equivalent descriptions of the same decision problem can yield systematically different responses—a pattern of results known as framing (or context) effects. Various different types of framing have been examined in the literature including risky-choice (or loss vs. gain) framing [14], attribute framing [15], and goal (or message) framing [16] to name a few (see [17], [18] for reviews). However, most germane to the current investigation is a form of framing that has been shown to interact with loss aversion, such that a loss is perceived as less aversive when cast in one frame than when cast in another. To elaborate, it has been shown that a negative outcome that is framed as a foregone-gain—a possible gain that is relinquished or attenuated—is perceived as fairer and less painful than an objectively equivalent framed actual loss [10], [19]–[23]. To illustrate, Kahneman et al. [19] found that when a group of participants was asked to decide whether the addition of a $200 surcharge on a brand of car that is in short supply—perceived as an actual loss—is fair or unacceptable, most people (71%) deemed such an economic action to be unacceptable. By contrast, when a second group of participants was presented with an objectively equivalent scenario in which the surcharge was replaced by the removal of a $200 discount—perceived as a reduction of a gain—most people (58%) deemed such an economic action to be fair.

It follows from the above that people should be more willing to support emission cuts when the future costs of reducing emissions are framed as a foregone-gain. However, counter to this prescription, public communications about climate policy impacts typically frame the costs as a loss [13], although the extent of this communication bias has yet-to-be quantified. In order to provide an indication of the magnitude of this bias, the authors conducted a search of Australian newspaper articles for communications regarding the future costs of Australia's carbon pricing scheme. The search revealed that the use of a loss frame outweighed a foregone-gain frame by a ratio of 10∶1 (see Table 1) suggesting that the bias is significant and may be exerting a detrimental effect on support for carbon policy (see Methods for further details about the newspaper search).

**Table 1:** Communications regarding the future costs of Australia's carbon pricing scheme—commonly referred to as the “carbon tax”—identified by the newspaper search along with the frame (loss vs. foregone-gain) they are cast within and the source they originate from.

Frame-Type	Example	Source
Loss	*Opposition Leader Tony Abbott this week will argue that real wages will fall under a carbon tax. On the basis of analysing government statistics, he will say the carbon tax is expected to cause real wages to be almost 1 per cent lower than they would otherwise be by 2020. For someone on current average adult full-time wages of about $70,000 this would be equivalent to a cut in salary of about $600 a year*.	The Age, 6 February 2012
Loss	*“The carbon tax will cause a large and continually growing fall-off in GDP,” the note released last night said, suggesting cumulative output loss would be $32 billion by 2020 and top $1 trillion by 2050 in real 2010 dollars*.	The Daily Telegraph, 6 February 2012
Loss	*The Centre for International Economics report quantifies this additional reduction in our national economic output as an extra $30 billion across six years, taking the overall impact of the carbon tax on GDP close to $50bn by 2018*.	The Australian, 13 March 2012
Loss	*Yet despite those generous assumptions, the Government's carbon tax modelling suggests that by 2050 our GDP will be $100 billion less than it would have been without a carbon tax… The cumulative effect of the carbon tax on our GDP between now and 2050 will be a reduction in our economic growth to the tune of $1 trillion in today's dollars*.	The West Australian, 26 June 2012
Loss	*Relying on Treasury modelling of the carbon tax, the opposition will highlight the reduction of wage growth and the economic impacts to families of the scheme… According to the opposition, these include a reduction in real wage growth by almost 1 per cent, translating to a salary reduction per year of $600 by 2020 and $4,000 by 2050 for a worker on current average adult full-time earnings*.	The Australian Financial Review, 6 February 2012
Loss	*He said [Tony Abbott at the Tasmanian Liberal Party Council] Treasury estimates showed Australians would be $5,000 worse off on average by 2050, while GDP would be reduced by $1 trillion*.	The Australian Financial Review, 27 August 2012
Frame-Type	Example	Media source
Loss	*Treasury's own modelling shows the carbon tax will erode GDP with a cumulative loss of output of $32 billion by 2020 rising to a staggering $1 trillion by 2050 in 2010 dollars*.	The Australian, 9 January 2013
Loss	*“Australia's annual GDP growth might only be 0.1 per cent lower every year with a carbon tax than without one but the cumulative loss in GDP between now and 2050 is $1 trillion.”*	The Australian, 3 September 2013
Loss	*Over the next six years the carbon tax will cost an average household more than $3000, including a whopping $900 a year by 2020*	The Daily Telegraph, 19 September 2013
Loss	*The carbon tax, according to the previous government's own modelling, will impose losses with a present value as high as 83 per cent of current Australian GDP, or $1.25 trillion*.	The Australian, 28 October 2013
Foregone-gain	*The Opposition Leader used his last appearance at the National Press Club before Saturday's election to attack the economic impact of the carbon tax, warning the cumulative loss in GDP of the policy between now and 2050 would be $1 trillion… But Climate Change Minister Mark Butler hit back, saying Treasury modelling showed the economy would be $41 trillion bigger in 2050 with a carbon price.*	The Australian, 3 September 2013

The first ten examples are couched within a loss frame, where the cost of reducing Australia's emissions is expressed as a decrease in national income/GDP relative to the baseline levels expected in 2020 and/or 2050. The final example is cast within a foregone-gain frame, where the cost of reducing Australia's emissions is expressed as a reduction in a gain in 2050.

A second potential solution to the problem of how to increase support for emission reductions is by using persuasive social-norming messages. Social norms refer to people's perceptions of how others behave in the relevant social context. A wealth of research indicates that social norms influence intentions and behaviour [24]–[26]. Thus, people tend to behave based on what they think others are doing. Accordingly, decisions such as whether to tip the waiter in a restaurant, how much alcohol to consume at a social gathering, and whether to give money to a charity are contingent, to some extent, on what we think most other people do in these situations. In the field of pro-environmental behaviours, there have been numerous demonstrations of the power of persuasive messages that are couched in terms of social norms to influence behaviour. For example, a normative message about average neighbourhood energy consumption has been shown to reduce energy use amongst households with above-average consumption [27]–[29]. Persuasive messages that make social norms salient have also been shown to influence littering [24], recycling [30], and environmental conservation amongst hotel guests [31], [32]. By implication, social norms may also be an effective tool for leveraging support for emission cuts.

In summary, we are interested in how framing and persuasive normative messages influence Australians GHG emission policy preferences. Study 1 examined whether framing the future costs of reducing Australia's CO_2_ emissions as a foregone-gain increases people's support for emission-reduction policies, compared to when objectively identical costs are framed as a loss. It also examined whether normative messages identifying Australia as one of the world's most prolific CO_2_ emitters further boosts support for emission reduction. Study 2 incorporated the same framing manipulation as Study 1 in conjunction with a normative message about the emission policy preferences of participants from the first study.

## Study 1: Framing and normative messages regarding Australia's CO_2_ emissions

In Study 1, university students (*N* = 120) were initially given a text passage highlighting the link between CO_2_ emissions and climate change. Participants were then randomly assigned to one of three social norm conditions (control vs. average-norm vs. rank-norm) and given further information about CO_2_ emissions (based on grams of CO_2_ emitted per kWh from electricity and heat generation). Participants in the control condition were given information about Australia's CO_2_ emissions per kWh; participants in the average-norm condition were given a normative message highlighting that Australia's CO_2_ emissions per kWh are significantly above the world average; whilst participants in the rank-norm condition were given a normative message that identified Australia as the 5th largest emitter of CO_2_ per kWh out of 139 countries. The average-norm and rank-norm conditions thus made salient the social norm that other countries are doing more to manage their CO_2_ emissions than Australia but the rank-norm condition made the extent of this departure from the norm more salient.

**Figure 1:**
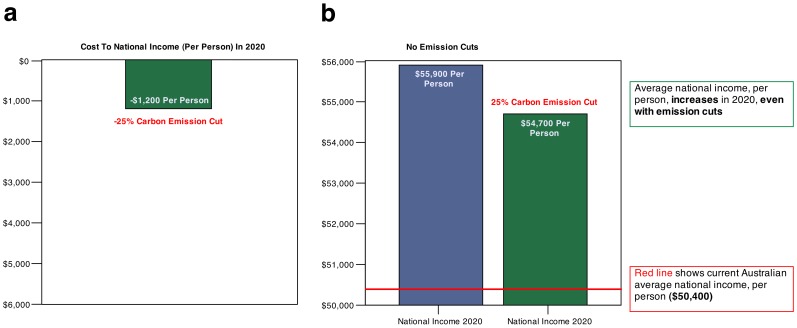
Examples of the graphical interfaces used in the two framing conditions in Study 1. In the loss condition (a), the graphical interface contained a bar chart with a single inverted green bar showing the cost to Australian average national income under different emission cut scenarios (25% in the example). Thus, the loss frame emphasised that in the presence of emission cuts national income will decrease from the baseline levels expected for 2020. In the foregone-gain condition (b), the graphical interface contained a bar chart with a blue bar showing the average national income in 2020 in the absence of emission cuts; a green bar showing the expected average national income under the different emission cut options (25% in the example); and a horizontal red line showing the current average national income of $50,400. Thus, the foregone-gain frame emphasised that under emission cuts national income will rise from current levels by 2020 but not by as much as in the absence of emission cuts.

Participants were subsequently asked to indicate their policy preferences regarding how Australia should manage its CO_2_ emissions using a graphical interface. The interface displayed the cost to average national income (per person) in 2020 of reducing Australia's current emissions by different amounts (ranging from 0% to 50%, in steps of 5%). Participants were randomly assigned to one of two framing conditions (loss vs. foregone-gain), which differed in terms of how they conveyed the numerically identical costs (which were based on real economic modelling released by the Australian Treasury [33]; see Methods for more detail). The loss graphical interface highlighted that in the presence of emission cuts national income would decrease from the baseline levels expected for 2020 (Fig. 1a). By contrast, the foregone-gain graphical interface underscored that under emission cuts average national income would rise from current levels in 2020, but not by as much as in the absence of emission cuts (Fig. 1b). Starting from an emission cut of 0%, participants were required to adjust the emission cut displayed in the graphical interface to a level that they would be willing to accept to reduce the risk of climate change.

We anticipated a main effect of social norm, with larger emission cuts in the average-norm than the control condition, and with larger emission cuts in turn in the rank-norm than the average-norm condition. The latter expectation was derived from recent studies highlighting that people may be more sensitive to ordinal rank than average-only social-norming messages [34]–[36]. We also anticipated a main effect of framing, with larger emission cuts in the foregone-gain than the loss condition.

**Figure 2:**
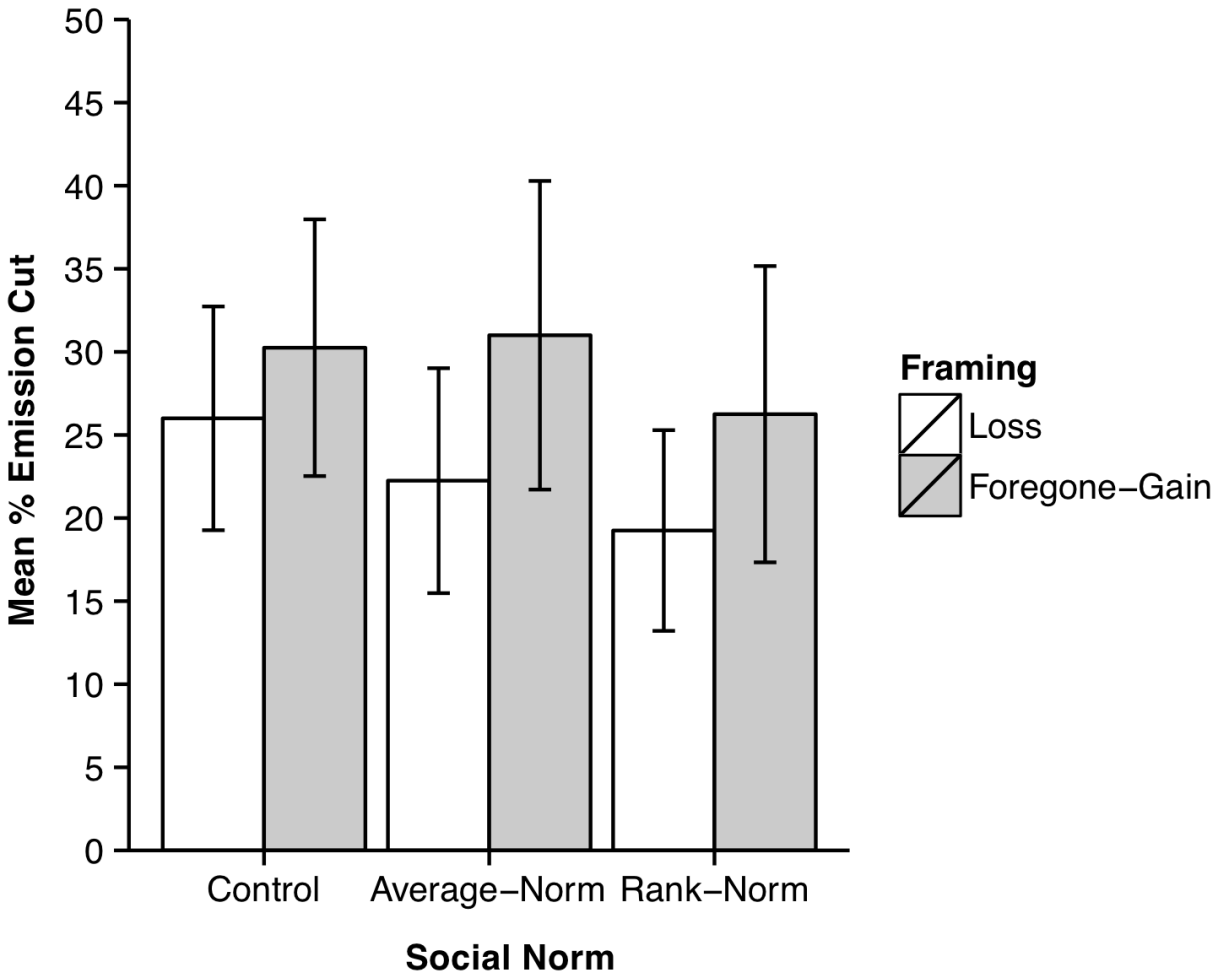
Mean emission cuts in Study 1 as a function of the framing and social norm manipulations. Error bars show 95% confidence intervals.

Fig. 2 shows the observed mean emission cuts across the six conditions. Averaging over conditions, the mean cut was 26%, indicating considerable willingness to reduce Australia's emissions. The data were analysed using a 2 (framing: loss vs. foregone-gain) ×3 (social norm: control vs. average-norm vs. rank-norm) between-participants Analysis of Variance (ANOVA [37]) performed using the anova function from the CAR package in the statistical computing software R (used for all subsequent statistical analyses). Effect size estimates are provided—for focused comparisons only—using Pearson's *r*. There was a significant main effect of framing, *F*(1,114) = 4.965, *p* = .028, *r* = .204, with larger emission cuts in the foregone-gain than the loss condition, no significant main effect of social norm, *F*(2,114) = 1.146, *p* = 0.322, and no significant interaction, *F*(2,114) = .192, *p* = .826.

Study 1 revealed that framing the costs of policy action as a foregone-gain increased the amount by which people were prepared to reduce Australia's emissions, compared to when framed as a loss. However, the social-norming messages did not have the anticipated effect (they may even have backfired; see Discussion). This may be because the normative feedback pertained to the behaviour of people from other countries, who are likely to be perceived as an out-group, whereas in most social-norming studies the feedback pertains to the behaviour of a perceived in-group consisting of one's peers (e.g., [24], [29]–[31]). Accordingly, Study 2 incorporated a more typical social-norming manipulation in which participants were given normative feedback vis-á-vis the emission policy preferences of an ostensible peer group—viz. participants from Study 1. It also sought to replicate the framing effect in Study 1 using a larger and more representative sample of Australian respondents.

## Study 2: Framing and normative messages about others' emission policy preferences

Participants (*N* = 1,200) were randomly assigned to a no-norm condition in which no normative message was provided or to a with-norm condition in which normative information about the emission policy preferences of participants from Study 1 was conveyed within the graphical interfaces. Note that since participants in Study 2 were on average twice as old as those in Study 1 and from a different demographic (see Methods), to ensure that individuals in the with-norm condition nevertheless perceived them as peers, the only information revealed about the sample was that they were Australian residents who had taken part in a similar study of emission policy preferences. Within the no-norm and with-norm groups, half of the participants were randomly assigned to the loss condition, the other half to the foregone-gain condition.

**Figure 3:**
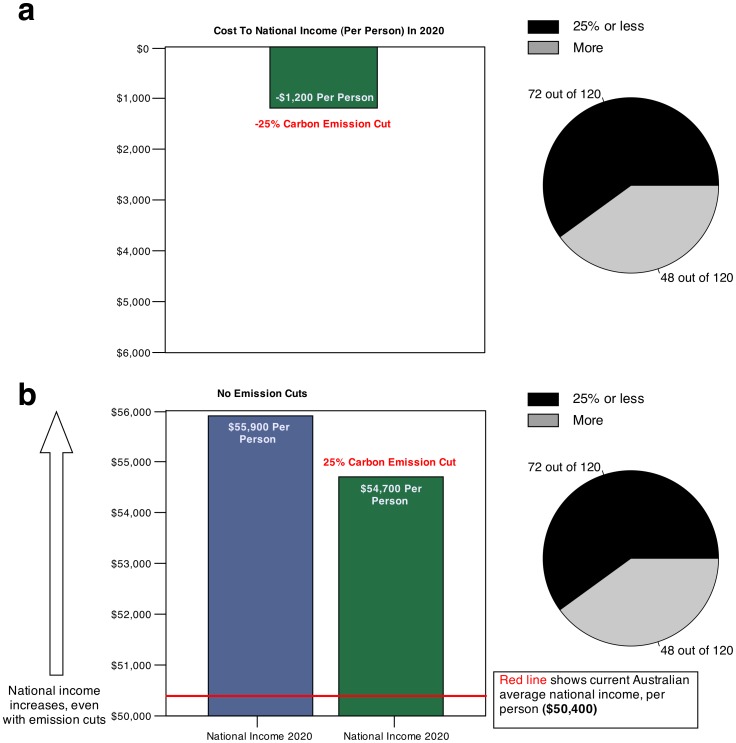
Examples of the loss (a) and foregone-gain (b) versions of the graphical interfaces employed in the with-norm condition of Study 2. The graphical interfaces were similar to those employed in Study 1 but with the addition of a pie chart illustrating the emission policy preferences of participants in that study, relative to the emission cut option displayed in the bar chart. See main text or Methods for more details.

The graphical interfaces for the no-norm condition were similar to those employed in Study 1. However, the interfaces were redesigned for the with-norm condition. The interfaces were augmented with a pie chart that conveyed information about the number of people in our initial study that reduced emissions by the amount shown in the bar chart or less, and the number that chose to reduce emissions by more. For example, Fig. 3a and Fig. 3b show screenshots of the loss and foregone-gain graphical interfaces, respectively, in the with-norm condition for a 25% emission cut, from which it can be seen that 72 out of 120 people in the previous study chose to reduce emissions by this amount or less, whereas 48 out of 120 people chose to reduce emissions by more than 25%. When the participant adjusted the emission cut shown in the bar chart, the information in the pie chart was updated to reflect the new values based on the pattern of responses of participants in Study 1. Table 2 shows the pie chart values that were communicated to participants for the different emission cut scenarios in the with-norm condition. It can be seen from inspection of this table, for example, that if the participant increased the emission cut to 30%, the pie chart showed that 79 out of 120 people in Study 1 chose to reduce emissions by this amount or less, whereas 41 out of 120 people chose to reduce emissions by more than 30%. As in Study 1, participants were required to adjust the emission cut displayed in the graphical interface to a level that they would be willing to accept to reduce the risk of climate change.

**Table 2:** Social-norming information communicated to participants in the with-norm condition of Study 2.

Emission cut (*x*)	*x* or less	More than *x*
0%	7 out of 120	113 out of 120
5%	15 out of 120	105 out of 120
10%	28 out of 120	92 out of 120
15%	48 out of 120	72 out of 120
20%	62 out of 120	58 out of 120
25%	72 out of 120	48 out of 120
30%	79 out of 120	41 out of 120
35%	86 out of 120	34 out of 120
40%	91 out of 120	29 out of 120
45%	92 out of 120	28 out of 120

The table shows for each emission cut option (column 1), the number of people in Study 1 who chose to reduce emissions by this amount (*x*) or less (column 2), and the number of people who chose to reduce emissions by more than this amount (column 3). These values were communicated to participants in the with-norm condition of Study 2 via the pie chart incorporated into the graphical interfaces for this condition (see Fig. 3). For example, for a 5% emission cut, the pie chart showed that in Study 1, 15 out of 120 people chose to reduce emissions by this amount or less, whereas 105 out of 120 people chose to reduce emissions by more than 5%. *Note*—for a 50% emission cut, the pie chart showed the number of people in Study 1 who chose to reduce emissions by this amount—viz. 28 out of 120—and the number of people who chose to reduce emissions by less than 50%—viz. 92 out of 120.

**Figure 4:**
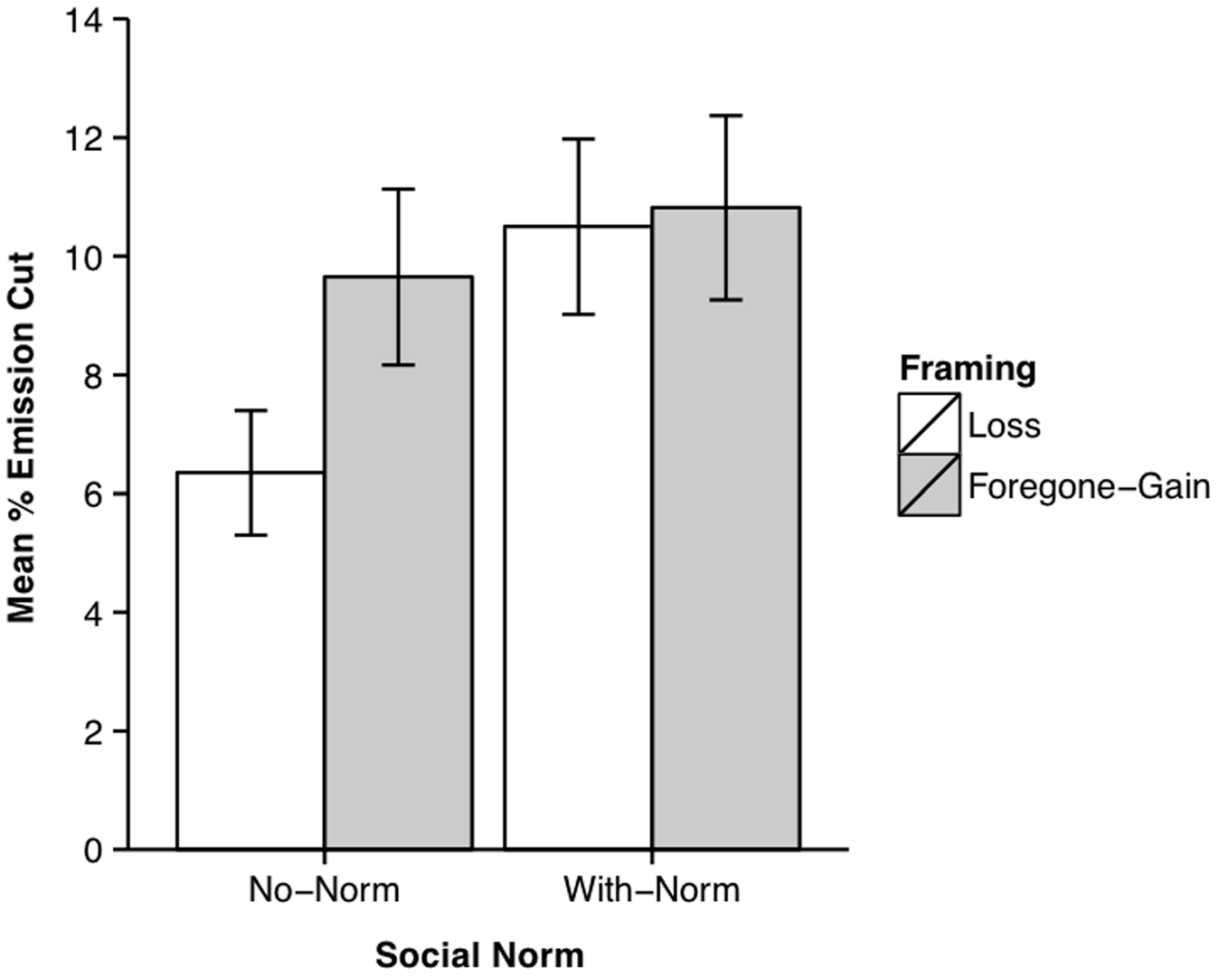
Mean emission cuts in Study 2 as a function of the framing and social norm manipulations. Error bars show 95% confidence intervals.

The data are shown in Fig. 4. Averaging over conditions, the mean emission cut was 9%, indicating that participants drawn from the general population were not prepared to reduce emissions by as much as the university students who participated in Study 1. Although the reason for this disparity is unclear, one speculation is that the sample of respondents in the current study was more heterogenous in terms of political views than the sample in Study 1, perhaps incorporating a greater proportion of political conservatives who are known to be less likely to accept that human GHG emissions are causing anthropogenic global warming than political liberals or moderates [38]–[42]. A 2 (framing: loss vs. foregone-gain) ×2 (social norm: no-norm vs. with-norm) ANOVA performed on the data revealed a significant main effect of framing, *F*(1,1196) = 6.438, *p* = .011, *r* = .073, with larger emission cuts in the foregone-gain than the loss condition, a significant main effect of social norm, *F*(1,1196) = 13.904, *p*<.001, *r* = .107, with larger emission cuts in the with-norm than the no-norm condition, together with a significant interaction, *F*(1,1196) = 4.363, *p* = .037.

To scrutinise the interaction, a simple-effects analysis was performed. This revealed a significant effect of framing for the no-norm condition, *F*(1,1196) = 10.699, *p* = .001, *r* = .094, with larger emission cuts in the foregone-gain than the loss condition, but there was no significant effect for the with-norm condition, *F*(1,1196) = 0.101, *p* = .751, *r* = .009. There was also a significant effect of social norm for the loss condition, *F*(1,1196) = 16.921, *p*<.001, *r* = .118, with larger emission cuts in the with-norm than the no-norm condition, but there was no significant effect for the foregone-gain condition, *F*(1,1196) = 1.345, *p* = .246, *r* = .034.

Study 2 replicated Study 1 in showing an effect of framing—with larger emission cuts in the foregone-gain than in the loss condition—using a larger and more representative sample. However, the novel result was that a normative message—this time regarding the emission policy preferences of others within a presumed peer group—also increased the amount people were prepared to reduce Australia's emissions. Study 2 additionally revealed an interaction, which arose because the normative information increased emission cuts in the loss, but not in the foregone-gain, condition.

## Discussion

The studies confirm that it is possible to “nudge” [43] people to support higher levels of emission cuts by changing the way in which the accompanying costs are framed or through the provision of appropriately crafted social-norming messages.

The most robust finding from the current studies relates to the effect of the framing manipulation. Consistent with previous theorising and empirical research highlighting that foregone-gains are preferred over losses [10], [19]–[23], people supported higher levels of emission cuts when the costs were framed as a foregone-gain than when they were framed as a loss (the one exception to this pattern was in Study 2, where the framing effect was eliminated when combined with a normative message about the emission policy preferences of others). That framing costs as a foregone-gain increased the amount people were prepared to reduce emissions is noteworthy because public messages about climate policy impacts typically frame the costs of reducing emissions as a loss [13]—a pattern confirmed by our analysis of newspaper communications regarding the future costs of Australia's carbon pricing scheme. This communication bias is potentially undercutting efforts to elicit support for emission cuts in at least two ways. First, carbon policies are likely to be judged less fair when framed as a loss, compared to a foregone-gain [19]. Second, as a possible direct consequence of the communication bias, one in five Australians incorrectly think that reducing emissions will cause future incomes to fall below current levels and subscription to this belief is a powerful predictor of opposition to carbon policy [13]. Fortunately, the reference point bias can be countered by reframing the future costs of reducing emissions as a foregone-gain, thus underscoring that incomes will continue to rise—albeit more slowly—under emission cuts. Our results therefore suggest that if climate policy communicators want to raise levels of support for carbon policies then they would be wise to frame the associated costs as a foregone-gain.

The results of the social-norming manipulations were more mixed. In Study 1, normative messages that Australia's emissions are above the world norm did not increase the amount people were prepared to reduce emissions. Indeed, there is some tentative evidence that the normative messages may have backfired. Specifically, in the loss condition, mean emission cuts were actually higher in the control than the average-norm condition, which in turn were higher than in the rank-norm condition (although the differences were not reliable, possibly due to statistical power—viz. the ability to reject the null hypothesis—being low owing to the relatively small sample size compared with Study 2). It has been noted that normative messages can backfire when they depict a behaviour as undesirably frequent [44]. Although our normative messages made salient that the social norm of most other countries is low-emissions-intensity power generation, they also inadvertently made salient that the social norm in Australia is high-emissions-intensity power generation. Since people tend to conform to the norms of groups with which they share a social identity [45] it is possible that people were influenced by the latter norm when making their policy choices. The results of Study 1 thus counsel that normative messages should be crafted with caution so that they do not accidentally depict high levels of engagement in the undesirable behaviour one is trying to change, and that the normative referent group should correspond to a perceived in-group rather than out-group.

Our second study circumvented both of these difficulties by presenting people with normative information highlighting the willingness of other Australians to reduce their country's emissions. Specifically, normative information about the emission policy preferences of respondents in our first study successfully increased emission cuts in the loss condition. However, it did not influence emission policy preferences in the foregone-gain condition, consequently abolishing the effect of framing. How to explain this result? Studies have shown that perceived cost fairness is an important predictor of an individuals willingness-to-pay for a good or service [46]–[48]. This implies that when making decisions about how much to pay, there is a fair reference point about which gains and losses are evaluated. In the current studies, this reference point might coincide with the maximum emission cut that an individual is willing to pay for. Reframing the costs as a foregone-gain may therefore have raised an individual's fair reference point to an upper bound, rendering the social-norming message impotent, because people are generally unwilling to be nudged beyond this reference point. This putative explanation raises the intriguing possibility that attempts to shift people's reference point—via anchoring for example [49]–[51]—might increase the amount people are prepared to reduce emissions further still.

A final issue merits comment; namely, the relative effectiveness of the framing and social-norming interventions as communication devices. The impact of the two interventions in Study 2 was comparable, suggesting that they are equally effective at inducing support for emission cuts. However, their effects were not additive—we did not find evidence to support the contention that communications integrating both approaches would have a greater impact than either approach employed in isolation. We suggest therefore that either approach can be used independently as a device to build support for carbon policies, but there is little precedent for using both simultaneously. We further note that one shortcoming of the social-norming approach is that it is contingent upon the existence of a social norm of support for high levels of emission cuts within the target population. If this normative standard does not exist then the use of a social norm approach is precluded. By contrast, the framing approach has wider generality because all that is required is a straightforward reformulation of the impact of reducing emissions on future incomes.

As noted in the Introduction, loss aversion and status quo bias constitute formidable psychological barriers to efforts to galvanise support for emission reductions. However, our results show that the deleterious effect of such cognitive biases can be attenuated by reframing the costs of reducing emissions as a foregone-gain or by deploying persuasive social-norming messages. These findings complement those of other recent studies that have identified additional variables that are effective in inducing support for climate-mitigation measures. Those additional variables include framing the consequences of climate-mitigation in terms of gains rather than losses [52], highlighting the benefits to human health of tackling climate change [53], [54], and, notably, underscoring the pervasive scientific consensus that humans are responsible for global warming [55], [56]. Accordingly, climate change communicators now have at their disposal a range of methods for leveraging support for climate-mitigation policies.

## Methods

### Newspaper search

On January 29, 2014, we searched Factiva—an online research tool for accessing content from different sources (viz. newspapers, journals, and magazines)—for newspaper articles containing statements regarding the future costs of Australia's carbon pricing scheme—commonly referred to as the “carbon tax”. We used the search terms *cost near5 carbon tax*, *gain same carbon tax*, *GDP same carbon tax*, *grow same carbon tax*, *income same carbon tax*, *loss same carbon tax*, *treasury modelling same carbon tax*, and *wages same carbon tax* (note that the operator “same” identifies instances where two key words appear in the same paragraph, whereas the operator “near5” identifies instances where two key words appear within 5 words of each other. The latter operator was used with the key words “cost” and “carbon tax” to keep the number of results manageable, since the “same” operator generated a large volume of hits). Our search was confined to articles published in major Australian news outlets (*Canberra Times, Daily Telegraph, Herald Sun, Sun Herald, The Age, The Australian, The Australian Financial Review, The Sydney Morning Herald, The West Australian, and the Australian Broadcasting Corporation*) between 01/01/2012 and 29/01/2014. Using these criteria yielded 1,481 articles (including duplicates) the majority of which focused on the immediate credits or debits of the carbon tax for families and industry, rather than the projected future costs to Australian incomes and/or GDP. After elimination of these irrelevant articles, 11 remained of which 1 was deemed by the authors to have used a foregone-gain frame, with the remaining 10 deemed to have used a loss frame (see Table 1).

### Empirical studies

Both studies were conducted as part of larger surveys, which included additional items. Since those extra items appeared after those used in the studies described in this article—and could not therefore have influenced people's responses to the items that are relevant here—we do not describe them. Study 1 data were collected during April–August 2012 in the Cognitive Science Laboratory at the University of Western Australia using a university student sample. Participants read an information sheet initially outlining the nature of the research before providing written informed consent. The design, method, and consent procedure was evaluated and approved by the Human Research Ethics Office of the University of Western Australia which recognises 17 year old students attending the university as adults capable of providing their own informed consent. Study 2 data were collected by Qualtrics—a company that specialises in representative internet surveys—during June–July 2013 using a web-based community sample. The study was prefixed by an information sheet outlining the purpose of the research. Participants indicated their informed consent by mouse-clicking on a radio button to proceed to the survey questions. The design, method, and consent procedure was evaluated and approved by the Human Research Ethics Office of the University of New South Wales. All participant data in both studies were anonymised.

#### Study 1

Participants (*N* = 120; mean age  = 19.73; *SD* = 5.28; range  = 17–53; females  = 67%) were students in the School of Psychology at the University of Western Australia who took part in the study in exchange for course credits. The study employed a 2 (framing: loss vs. foregone-gain) ×3 (social norm: control vs. average-norm vs. rank-norm) between-participants design. Participants were allocated at random to one of the six conditions. The study was executed on a PC running Windows XP using a MATLAB programme designed with the Psychophysics Toolbox Version 3 (PTB-3) [57]–[59].

All participants were initially given a short text passage highlighting the link between human CO_2_ emissions and climate change. The passage included two accompanying graphics, one showing the increase in global CO_2_ emissions between 1890–2010, the second showing the increase in world land–ocean temperature over the same period. Next, participants were given information about International Energy Agency Estimates of grams of CO_2_ emitted per kWh from electricity and heat generation for Australia and other countries between 2005 and 2009 [60]. Participants in the control condition were given a text passage providing information about Australia's CO_2_ emissions per kWh only. Participants in the average-norm condition were given a text passage and accompanying graphic highlighting that Australia's CO_2_ emissions per kWh are significantly above the world average. Participants in the rank-norm condition were given a text passage and accompanying graphic identifying Australia as the world's fifth largest emitter of CO_2_ per kWh out of 139 countries.

Participants were subsequently asked to indicate their policy preferences regarding how Australia should manage its CO_2_ emissions using a graphical interface. The interface displayed the cost to average national income (per person) in 2020 of reducing Australia's current emissions by different amounts (ranging from 0% to 50%, in steps of 5%). In the loss condition (Fig. 1a), the graphical interface contained a bar chart with a single inverted green bar showing the cost to Australian average national income under the different emission cut options (25% in the example). In the foregone-gain condition (Fig. 1b), the graphical interface contained a bar chart with a blue bar showing the expected average national income in 2020 in the absence of emission cuts; a green bar showing the expected average national income under the different emission cut options (25% in the example); and a horizontal red line showing the current average national income of $50,400. Starting from an emission cut option of 0%, participants in both conditions were instructed to adjust the green bar—using the “up” and “down” arrow keys on the computer keyboard—to a level that they would be willing to accept to reduce the risk of climate change, before pressing the “enter” key to register their preferred policy option.

The emission reduction policy options conveyed to participants were based on real economic modelling released by the Australian Treasury [33]. However, the Treasury modelling only considered emission cuts of 5%, 10%, 15%, and 25%. To derive national income predictions for a 20% emission cut, and for emission cuts between 30–50% we took the predicted costs to national income in 2020 for each emission cut considered in the Treasury modelling and fit a linear regression model to those data. The resulting regression slope and intercept parameters were then used to extrapolate the costs to national income under the additional emission cut scenarios.

#### Study 2

The study employed a 2 (framing: loss vs. foregone-gain) ×2 (social norm: no-norm vs. with-norm) between-participants design. Participants (*N* = 1,200; mean age  = 44.38; *SD*  = 16.53; range  = 18–82; females  = 51%) were randomly assigned to one of the four conditions. The age and gender distribution of participants in each condition conformed to that of the most recent census report of the Australian Bureau of Statistics (this was not a coincidence; the authors specifically requested these distributions from the Qualtrics panels service responsible for data collection). The study was conducted using Qualtrics software (developed by Qualtrics)—a web-based survey software tool for the creation of survey instruments, distribution of surveys, data collection, storage and analysis.

Participants were initially provided with training in how to interpret the graphical interfaces used to convey the social-norming information in the with-norm condition. Participants were informed about a fictional study—unrelated to the emission reduction preference question—in which 800 Australians were asked how much they would be willing to reduce their household energy consumption, either 5%, 10%, 15%, or 25%. They were then shown four pie charts—one by one—graphically illustrating for each energy cut option, the number of people that chose to reduce their energy consumption by that amount or less, and the number that chose to reduce their energy consumption by more. Two of the graphics were accompanied by comprehension questions to ensure that participants could understand the information being conveyed. The purpose of this initial scenario was to give people practice at interpreting the similar pie charts that were subsequently used in the graphical interfaces to deliver the social-norming information in the with-norm condition. For consistency, participants in the no-norm condition were also exposed to this training scenario.

Participants were subsequently given the text passage and accompanying graphics linking human CO_2_ emissions to climate change used in Study 1, before being asked to indicate their policy preferences regarding how Australia should manage its CO_2_ emissions. The loss and foregone-gain graphical interfaces for the no-norm condition were similar to those used in Study 1, whereas the interfaces were refashioned for the with-norm condition. Specifically, the interfaces were augmented with a pie chart that conveyed information about the number of people in our initial study that chose to reduce emissions by the amount shown in the bar chart or less, and the number that chose to reduce emissions by more. For example, Fig. 3a and Fig. 3b show screenshots of the loss and foregone-gain graphical interfaces, respectively, in the with-norm condition for a 25% emission cut, from which it can be seen that 72 out of 120 people in the previous study chose to reduce emissions by this amount or less, whereas 48 out of 120 people chose to reduce emissions by more than 25%. When the participant adjusted the emission cut option shown in the bar chart, the information in the pie chart was updated to reflect the new values based on the pattern of responses of participants in Study 1. The pie chart values that were communicated to participants for the different emission cut options are displayed in Table 2.

Unlike Study 1, participants used a series of radio buttons beneath the graphical interface to navigate through the different emission cut options and indicate their preferred policy choice. For example, when the bar chart showed a 0% cut in emissions there were two radio buttons corresponding to “Reduce emissions by 0%” and “Reduce emissions by more”. If the participant selected the latter radio button the bar chart for a 5% emission cut was displayed along with three radio buttons corresponding to “Reduce emissions by less”, “Reduce emissions by 5%”, and “Reduce emissions by more”. Selecting the first radio button took the participant back to the bar chart for a 0% emission cut, selecting the second registered a 5% emission cut as their preferred policy choice and exited the graphical interface, whereas selecting the third button displayed the bar chart for a 10% emission cut.

## Supporting Information

S1 Text Passage **Introductory text passage on carbon emissions and climate change used in Studies 1 and 2.**
(PDF)

S2 Text Passage **Text passage for control condition in Study 1.**
(PDF)

S3 Text Passage **Text passage for average-norm condition in Study 1.**
(PDF)

S4 Text Passage **Text passage for rank-norm condition in Study 1.**
(PDF)

S1 Data **Raw data for Studies 1 and 2.**
(XLSX)
