# Supplementary material for: The Effect of Framing and Normative Messages in Building Support for Climate Policies
Source: PLoS One. 2014 Dec 15;9(12):e114335. doi: 10.1371/journal.pone.0114335 (PMC4266503; doi:10.1371/journal.pone.0114335)
Supplement: S1 Text Passage — Introductory text passage on carbon emissions and climate change used in Studies 1 and 2. (PDF) [file pone.0114335.s001.pdf]

Carbon Emissions and Climate Change

Since the nineteenth century, human-induced carbon emissions—emissions created through the burning of fossil fuels, such as coal and oil—have increased exponentially, with the greatest increase in emissions occurring in the past sixty years (see Figure A). Coinciding with this increase in emissions, there has also been a corresponding increase in the Earth's temperature, since the same period (see Figure B).

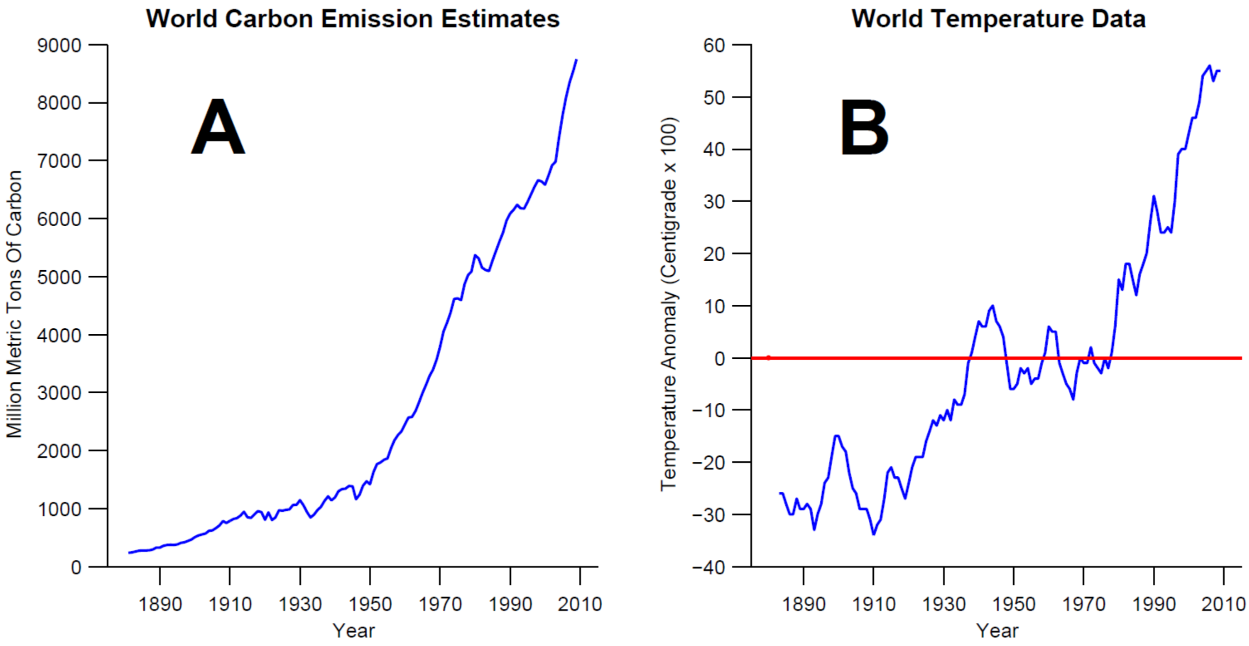

The scientific consensus is that human-induced carbon emissions are the principal determinant of this global warming. There is also a scientific consensus that unless global action is taken to mitigate the effects of climate change—through the reduction of human-induced carbon emissions—there will be notable adverse consequences for Earth's climate and its inhabitants.
