# Supplementary material for: The Effect of Framing and Normative Messages in Building Support for Climate Policies
Source: PLoS One. 2014 Dec 15;9(12):e114335. doi: 10.1371/journal.pone.0114335 (PMC4266503; doi:10.1371/journal.pone.0114335)
Supplement: S2 Text Passage — Text passage for control condition in Study 1. (PDF) [file pone.0114335.s002.pdf]

Text Passage S2.

According to the International Energy Agency (IEA), between 2005 and 2009, Australia's level of CO_2 emissions per unit of power generated (measured in grams of carbon dioxide per kilowatt-hour—gCO_2 per kWh) was 884gCO_2 per kWh.
