# Supplementary material for: The Effect of Framing and Normative Messages in Building Support for Climate Policies
Source: PLoS One. 2014 Dec 15;9(12):e114335. doi: 10.1371/journal.pone.0114335 (PMC4266503; doi:10.1371/journal.pone.0114335)

Text Passage S3.

According to the International Energy Agency (IEA), between 2005 and 2009, Australia emitted more than the world's average amount of CO2 per unit of power generated (measured in grams of carbon dioxide per kilowatt-hour—gCO2 per kWh).

Australia emitted 884gCO2 per kWh compared to a global average of 457gCO2 per kWh (see figure).

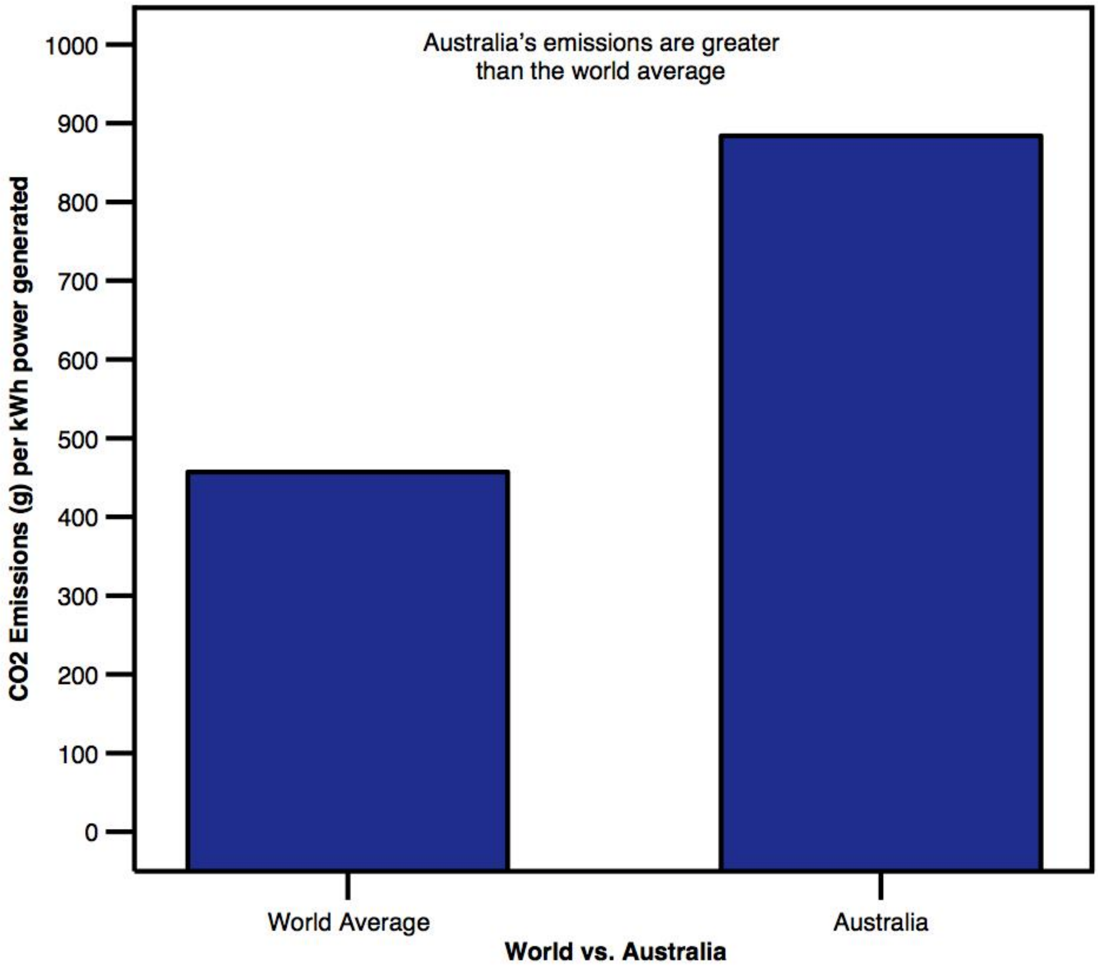

Supplement: S3 Text Passage — Text passage for average-norm condition in Study 1. (PDF) [file pone.0114335.s003.pdf]
