# Supplementary material for: The Effect of Framing and Normative Messages in Building Support for Climate Policies
Source: PLoS One. 2014 Dec 15;9(12):e114335. doi: 10.1371/journal.pone.0114335 (PMC4266503; doi:10.1371/journal.pone.0114335)
Supplement: S4 Text Passage — Text passage for rank-norm condition in Study 1. (PDF) [file pone.0114335.s004.pdf]

According to the International Energy Agency (IEA), between 2005 and 2009, Australia emitted more CO2 per unit of power generated (measured in grams of carbon dioxide per kilowatt-hour—gCO2 per kWh) than 134 out of 139 countries (see figure).

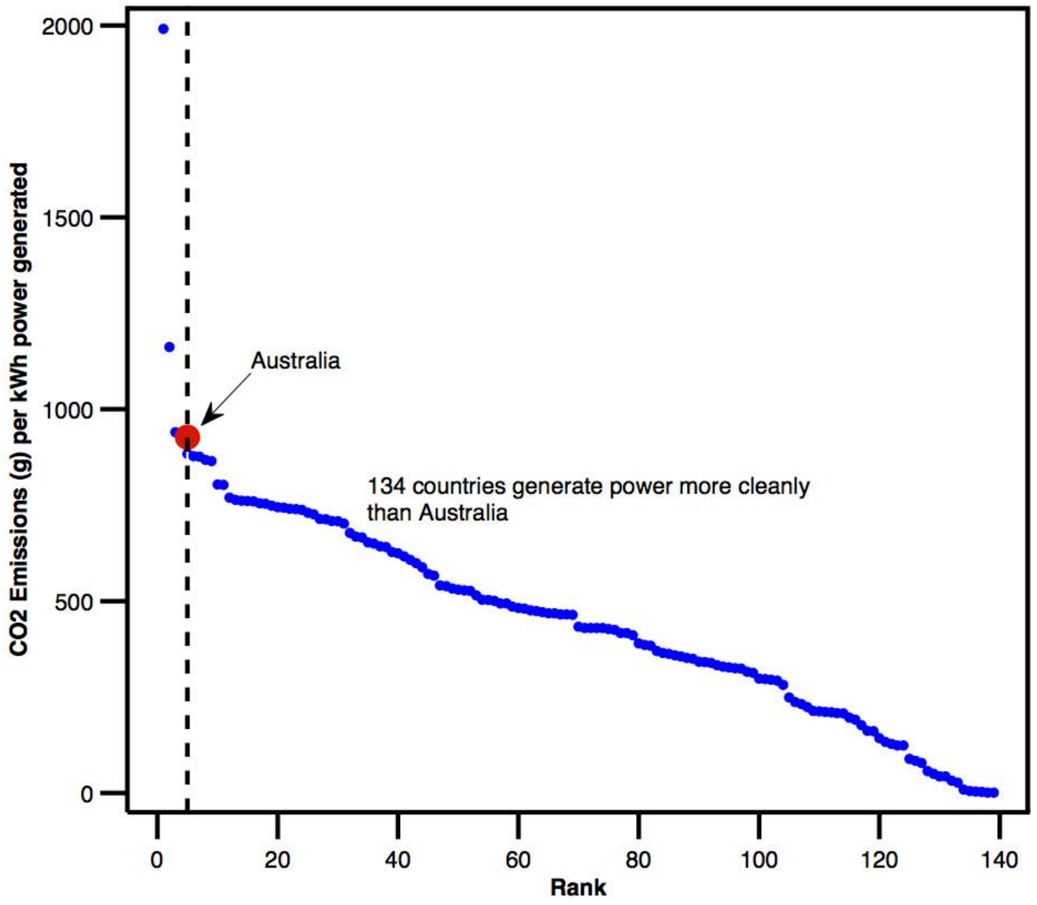

Australia emitted 884gCO2 per kWh, making it the world's **5th-largest** carbon emitter. Only four countries emitted more CO2 than Australia:

- Botswana (1991gCO2 per kWh),
- Cambodia (1162gCO2 per kWh),
- Malta (940gCO2 per kWh), and
- India (939gCO2 per kWh).

All other OECD countries emitted less than Australia (457gCO2 per kWh on average)
